# Enrofloxacin and Macrolides Alone or in Combination with Rifampicin as Antimicrobial Treatment in a Bovine Model of Acute *Chlamydia psittaci* Infection

**DOI:** 10.1371/journal.pone.0119736

**Published:** 2015-03-13

**Authors:** Annette Prohl, Markus Lohr, Carola Ostermann, Elisabeth Liebler-Tenorio, Angela Berndt, Wieland Schroedl, Michael Rothe, Evelyn Schubert, Konrad Sachse, Petra Reinhold

**Affiliations:** 1 Institute of Molecular Pathogenesis at Friedrich-Loeffler-Institut (Federal Research Institute for Animal Health), Naumburger Str. 96a, 07743 Jena, Germany; 2 Institute of Bacteriology and Mycology, Veterinary Faculty at The University of Leipzig, Leipzig; An den Tierkliniken 29, 04103 Leipzig, Germany; 3 LIPIDOMIX GmbH, Robert-Rössle-Str.10, Haus 55, 13125 Berlin, Germany; 4 OIE Reference Laboratory for Chlamydiosis at Friedrich-Loeffler-Institut (Federal Research Institute for Animal Health), Naumburger Str. 96a, 07743 Jena, Germany; ithree Institute, AUSTRALIA

## Abstract

*Chlamydia psittaci* is a zoonotic bacterium with a wide host range that can cause respiratory disease in humans and cattle. In the present study, effects of treatment with macrolides and quinolones applied alone or in combination with rifampicin were tested in a previously established bovine model of respiratory *C*. *psittaci* infection. Fifty animals were inoculated intrabronchially at the age of 6–8 weeks. Seven served as untreated controls, the others were assigned to seven treatment groups: (i) rifampicin, (ii) enrofloxacin, (iii) enrofloxacin + rifampicin, (iv) azithromycin, (v) azithromycin + rifampicin, (vi) erythromycin, and (vii) erythromycin + rifampicin. Treatment started 30 hours after inoculation and continued until 14 days after inoculation (dpi), when all animals were necropsied. The infection was successful in all animals and sufficient antibiotic levels were detected in blood plasma and tissue of the treated animals. Reisolation of the pathogen was achieved more often from untreated animals than from other groups. Nevertheless, pathogen detection by PCR was possible to the same extent in all animals and there were no significant differences between treated and untreated animals in terms of local (i.e. cell count and differentiation of BALF-cells) and systemic inflammation (i.e. white blood cells and concentration of acute phase protein LBP), clinical signs, and pathological findings at necropsy. Regardless of the reduced reisolation rate in treated animals, the treatment of experimentally induced respiratory *C*. *psittaci* infection with enrofloxacin, azithromycin or erythromycin alone or in combination with rifampicin was without obvious benefit for the host, since no significant differences in clinical and pathological findings or inflammatory parameters were detected and all animals recovered clinically within two weeks.

## Introduction

Infections with the zoonotic bacterium *Chlamydia (C.) psittaci* can cause severe respiratory and systemic disease in humans. The original hosts of *C*. *psittaci* are birds, but it has also been detected in several mammalian species, including cattle, horses, sheep, and pigs, where it is often associated with respiratory and reproductive disease [1–4]. In cattle, *C*. *psittaci* is by far more widespread than formerly assumed and is now known to have a significant impact on livestock productivity [2,5–7]. A study detecting DNA of *C*. *psittaci* in conjunctival swabs of human and bovine samples from the same farms suggested that transmission from cattle to human or vice versa is possible [8]. Therefore, chlamydiosis in cattle is not only an economic problem, but might also become a public health issue.

The pathogenesis of chlamydiosis has been subject to intense research in the last years (reviewed by Knittler and colleagues [9]), but reasons for the failure of successful treatment of chlamydial infections in both humans and animals are only suspected in most cases.

When it comes to treatment of chlamydial infections in various species, the drugs of choice are tetracyclines in most cases [10]. In birds, successful doxycycline treatment of experimental and naturally occurring infections with *C*. *psittaci* has been reported [11,12], whereas no sufficient impact of doxycycline treatment on the clinical outcome or severity of inflammation in an experimentally induced respiratory *C*. *psittaci* infection in calves could be shown [13]. Recent studies in *C*. *trachomatis*-infected humans reported cure rates of 90% or higher after doxycycline treatment [14,15]. However, most studies in humans should be interpreted very carefully, since a single follow-up screening for chlamydial DNA or RNA is not suitable for determining the actual infection state [16].

In pigs, some *C*. *suis* strains have developed tetracycline resistance [17–19] and it has been shown *in vitro* that the development of drug-resistant *C*. *psittaci* strains is possible [20,21]. Right now, antimicrobial resistance is not assumed to be a problem in chlamydiae other than *C*. *suis*, but field isolates are only very rarely screened for antimicrobial resistance in routine diagnostic procedures. Therefore, the actual situation can only be roughly assessed [22].

Alternative treatments include macrolides and quinolones, which are also frequently used in human [23,24] and veterinary medicine [25,26] to treat different chlamydial infections. Both are able to reach sufficiently high intracellular levels necessary for elimination of the obligate intracellular chlamydiae. It was shown in a cell culture model of an acute and a persistent C. psittaci infection that adding rifampicin increases the antichlamydial effect of macrolides and quinolones [27]. This new therapeutic approach was now intended to be applied to the situation in a living host. For this, we chose a previously established and well-characterized bovine model of acute respiratory infection with *C*. *psittaci* [28–31]. Calves often acquire chlamydial infections in field settings and therefore represent a frequent host to chlamydiae and, at the age of 6–8 weeks, they resemble adult humans in size and lung volumes. Also, in contrast to laboratory rodents, they offer the possibility of sampling in one and the same individual repeatedly to monitor the course of disease. After inoculation, the clinical picture resembles the course of acute human psittacosis, thus making calves a valuable model animal for this disease and allowing results to be beneficial for both human and veterinary medicine.

The present controlled and partially blinded prospective study was conceived to answer two questions: (i) What are the effects of treatment with macrolides or quinolones on the course of an acute respiratory *C*. *psittaci* infection in calves and (ii) can the results of the cell culture model, i.e. increased antichlamydial activity of macrolides and quinolones when combined with rifampicin, be transferred to the situation in the living frequent host and if so, is this clinically relevant?

## Results

### Antimicrobial levels

No significant levels of azithromycin, erythromycin, rifampicin, enrofloxacin and its active metabolite ciprofloxacin could be detected in blood plasma sampled 1 h prior to inoculation. Median and range of antibiotic levels in blood plasma and tissue for the different treatment groups from 3 days post inoculation (dpi) until 14 dpi are given in Table 1.

**Table 1:** Concentrations of antimicrobial substances in blood plasma and tissue samples.

		Rifampicin	Enrofloxacin		Enrofloxacin in combination with Rifampicin			Azithromycin	Azithromycin in combination with Rifampicin		Erythromycin	Erythromycin in combination with Rifampicin	
		Rifampicin	Enrofloxacin	Ciprofloxacin	Enrofloxacin	Ciprofloxacin	Rifampicin	Azithromycin	Azithromycin	Rifampicin	Erythromycin	Erythromycin	Rifampicin
**plasma**	time, dpi	[ng/mL]	[ng/mL]	[ng/mL]	[ng/mL]	[ng/mL]	[ng/mL]	[ng/mL]	[ng/mL]	[ng/mL]	[ng/mL]	[ng/mL]	[ng/mL]
	3	3053.0 (2728.0; 3907.0)	766.5 (461.0; 885.0)	141.0 (114.0; 195.0)	770.0 (445.0; 2363.0)	174.5 (105.0; 210.0)	3839.5 (1592.0; 5044.0)	68.0 (42.1; 145.3)	127.6 (71.0; 199.6)	2654.0 (1649.0; 4027.0)	268.3 (13.4; 852.0)	502.3 (245.9; 733.3)	2389.0 (1657.0; 4842.0)
	5	3220.5 (1902.0; 5140.0)	490.5 (327.0; 1321.0)	103.5 (61.3; 348.0)	364.0 (236.0; 847.0)	145.0 (89.0; 166.0)	3669.0 (1354.0; 5211.0)	103.7 (69.0; 154.9)^a^	151.9 (103.8; 245.1)^b^	3740.5 (1329.0; 6993.0)	431.6 (235.4; 585.3)	316.7 (144.7; 620.7)	3057.5 (582.0; 6773.0)
	7	1172.5 (827.0; 1983.0)	315.5 (104.0; 371.0)	86.65 (57.7; 310.0)	153.5 (105.0; 306.0)	82.45 (72.6; 87.4)	2074.0 (828.0; 4309.0)	154.6 (102.4; 225.1)	201.0 (151.1; 289.4)	2115.5 (389.0; 6211.0)	248.4 (134.4; 465.5)	323.3 (20.2; 596.3)	1246.5 (182.6 6643.0)
	10	1472.0 (977.0; 1968.0)	223.5 (151.0; 354.0)	75.0 (60.4; 216.0)	160.5 (72.9; 235.0)	88.25 (57.6; 140.0)	1275.5 (760.0; 2150.0)	192.5 (148.8; 355.4)	203.2 (173.4; 307.8)	1813.0 (420.0; 3437.0)	253.1 (95.6; 609.0)	315.2 (235.1; 478.9)	1056.5 (192.0; 2563.0)
	14	488.0 (315.0; 1457.0)	107.5 (45.0; 247.0)	54.05 (27.6; 78.1)	76 (28.9; 188.0)	47.75 (0.0; 81.4)	433.5 (133.0; 1298.0)	167.8 (141.8; 312.2)	202.2 (165.4; 399.5)	386.0 (117.0; 1516.0)	231.1 (9.2; 341.3)	271.0 (136.2; 420.3)	297.0 (77.5; 552.0)
**tissue**		[ng/g]	[ng/g]	[ng/g]	[ng/g]	[ng/g]	[ng/g]	[ng/g]	[ng/g]	[ng/g]	[ng/g]	[ng/g]	[ng/g]
lung	14	272.5 (122.0; 635.0)	119.0 (102.0; 326.0)	113.5 (0.0; 226.0)	0.0 (0.0; 272.0)	123.0 (0.0; 230.0)	165.0 (13.4; 851.0)	15994.9 (3584.3; 31123.6)	14427.0 (9823.2; 25318.6)	292.5 (62.1; 708.9)	324.2 (65.1; 793.6)^a^	821.1 (457.3; 1205.6)^b^	247.3 (83.0; 913.1)
muscle	14	222.5 (75.9; 365.0)	203.0 (0.0; 270.0)	129.0 (0.0; 239.0)	119.0 (0.0; 239.0)	125.0 (0.0; 159.0)	175.0 (52.9; 734.0)	625.3 (489.9; 923.8)	611.6 (395.7; 978.7)	230.4 (41.2; 376.5)	124.5 (23.1; 336.6)	353.0 (100.8; 557.5)	178.9; (66.4 462.0)
liver	14	10802.0 (7587.0; 13090.0)	301.5 (178.0; 764.0)	401.0 (272.0; 635.0)	219.5 (100.0; 515.0)	410.5 (173.0; 751.0)	10679.5 (5153.0; 16838.0)	23115.8 (12239.9; 53693.3)	14747.8 (6051.0; 25731.5)	16726.9 (7855.3; 35993.0)	702.3 (42.1; 1233.0)	1030.2 (573.5; 1842.3)	19778.8 (13172.7; 49231.1)

Values are given as median and range. Plasma levels of enrofloxacin, its active metabolite ciprofloxacin, erythromycin, and rifampicin were highest 3 and 5 dpi, whereas levels of azithromycin reached their maximum 10 dpi. Different letters (a, b) indicate statistically significant differences in antibiotic concentration in plasma and tissue between groups treated with or without rifampicin on the same day (*P* < 0.05, Mann-Whitney U-test).

Plasma levels of enrofloxacin, ciprofloxacin, erythromycin, and rifampicin reached their maximum shortly after the beginning of the treatment on 3 and 5 dpi. Azithromycin reached maximal plasma levels on 10 dpi.

Enrofloxacin concentrations in tissues (lung, liver, and muscle sampled 14 dpi) and in plasma (sampled 3, 5, 7, 10, and 14 dpi) were clearly lower in animals treated with enrofloxacin and rifampicin in combination than in animals treated with enrofloxacin alone, although this difference could not be statistically secured (Mann-Whitney U-test, *P* > 0.05). In four animals treated with both enrofloxacin and rifampicin, no measurable enrofloxacin concentrations were detected in the lung. In lung tissue of three out of these four animals, ciprofloxacin was detectable, only in one animal neither enrofloxacin nor ciprofloxacin could be detected. Concentrations of ciprofloxacin in tissue and plasma were comparably high in both groups treated with enrofloxacin.

Plasma levels of azithromycin were higher in animals treated with both azithromycin and rifampicin than in animals treated with azithromycin alone. This difference was only significant for 5 dpi (Mann-Whitney U-test, 5 dpi: *P* = 0.02; all other days: *P* > 0.05). Tissue levels of azithromycin were comparable in both groups treated with this drug.

In animals treated with erythromycin and rifampicin in combination, levels of erythromycin in plasma were slightly higher than in animals treated with erythromycin alone. Tissue concentrations of erythromycin were higher in animals receiving combination therapy than in animals receiving only erythromycin injections. For the concentration of erythromycin in lung tissue this was statistically significant (Mann-Whitney U-test, *P* = 0.01).

### Detection of the pathogen

#### Reisolation

In animals of all groups, reisolation of *C*. *psittaci* was possible from bronchial brushings obtained 4 dpi. Untreated controls and rifampicin-treated animals had most culture-positive specimens at that time point. From samples obtained 9 dpi, reisolation was possible in 4 untreated control animals and in one animal treated with erythromycin and rifampicin, and 14 dpi in one lung tissue sample from an untreated animal. Passaging of selected specimens of treated and untreated animals revealed that the pathogen was viable. It could be confirmed by DNA sequencing that the cultivated strain was the same as the strain DC15 used for inoculation of the animals. Numbers of culture-positive animals per group at the different time points are given in Table 2.

**Table 2:** Reisolation of Chlamydia psittaci from bronchial brushings.

	C	R	En	En + R	Az	Az + R	Ery	Ery + R
	(n = 7)	(n = 6)	(n = 6)	(n = 6)	(n = 7)	(n = 6)	(n = 6)	(n = 6)
**4 dpi**	4	4	2	2	1	1	2	2
**9 dpi**	4	0	0	0	0	0	0	1
**14 dpi**	1	0	0	0	0	0	0	0
total	9	4	2	2	1	1	2	3

Number of animals with positive reisolation results. Reisolation of *C*. *psittaci* was possible more often in untreated animals than in treated animals. C: untreated controls, R: rifampicin, En: enrofloxacin, En + R: enrofloxacin + rifampicin, Az: azithromycin, Az + R: azithromycin + rifampicin, Ery: erythromycin, Ery + R: erythromycin + rifampicin. dpi: days post inoculation.

#### Swabs, Tissue

**Fig 1:**
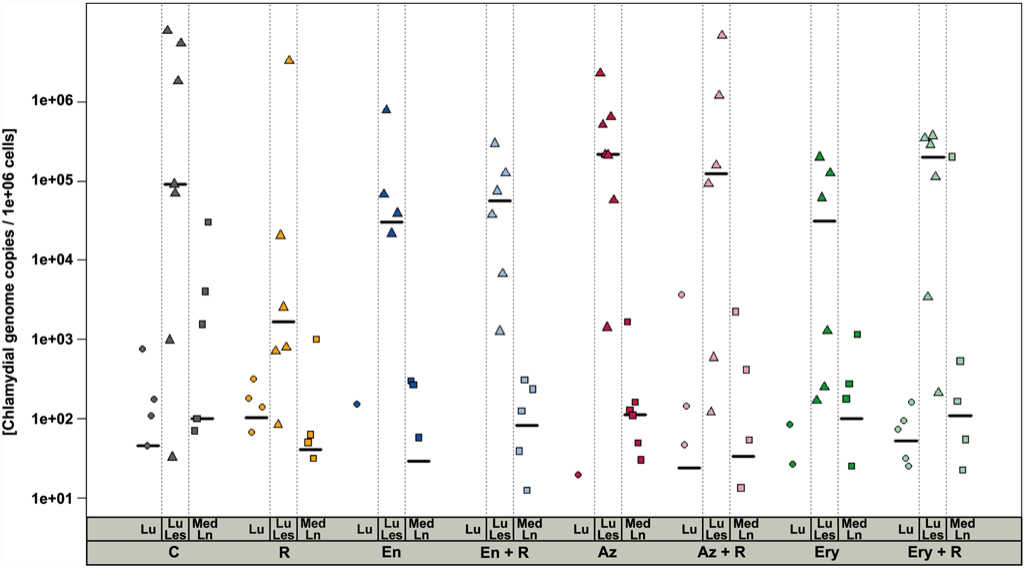
Chlamydial genome copies in tissue 14 days after inoculation. The number of chlamydial genome copies per one million cells determined with qrtPCR was higher in altered lung tissue (Lu Les, triangles) than it was in macroscopically normal lung tissue (Lu, circles) and in mediastinal lymph node (Med Ln, squares). Chlamydial DNA was detected in altered lung tissue of all animals except for two enrofloxacin-treated animals, whereas in some animals of all treatment groups no chlamydial DNA was detected in normal lung tissue and in the mediastinal lymph node. There was a high degree of variation in chlamydial genome copy numbers within the treatment groups and no statistically significant differences between treated and untreated groups for the amount of chlamydial DNA in the tissues sampled 14 dpi (many-to-one comparisons by Gao *et al*. (2008) with Hochberg-adjustment, *P* > 0.25). Black lines: medians, C: untreated controls, R: rifampicin, En: enrofloxacin, En + R: enrofloxacin + rifampicin, Az: azithromycin, Az + R: azithromycin + rifampicin, Ery: erythromycin, Ery + R: erythromycin + rifampicin. dpi: days post inoculation.

Chlamydial DNA was detected by qrt-PCR in normal and altered lung tissue and in pieces of the mediastinal lymph node sampled at necropsy 14 dpi in animals of all groups. The amount of detected genome copies did not differ statistically significant between treated and untreated animals (many-to-one comparisons by Gao *et al*. [32] with Hochberg-adjustment, *P* > 0.25) (Fig. 1).

DNA of *C*. *psittaci* was detected in pharyngeal swabs obtained 4 dpi in three animals treated with enrofloxacin, two animals treated with rifampicin and one animal treated with enrofloxacin and rifampicin. At 9 dpi, DNA of *C*. *psittaci* was detected in a pharyngeal swab of an animal treated with enrofloxacin and rifampicin and 14 dpi in the fecal swab of an untreated animal.

DNA of *C*. *psittaci* could not be detected in any other swabs.

#### Blood

None of the blood samples obtained during the study tested positive for chlamydial DNA.

### Clinical Score

Daily examination revealed the development of clinical signs of respiratory and general disease after inoculation with *C*. *psittaci* in all animals. The calves showed cough, raised body temperature, heart rate and respiratory rate, forced breathing, reduced appetite, swollen lymph nodes, and hyperemia of mucous membranes. Almost half of the clinical score was contributed by respiratory signs. The maximum of the clinical score was reached at 2 and 3 dpi. From there on, clinical health started to improve in all animals until 7 dpi, when the clinical score almost reached baseline level again. The rectal body temperature was maximal from 30 to 48 hours after inoculation (41.0°C; 39.9–42.0; median, range) and returned to physiological values until 6 dpi in all animals (38.7°C; 38.3–39.3).

**Fig 2:**
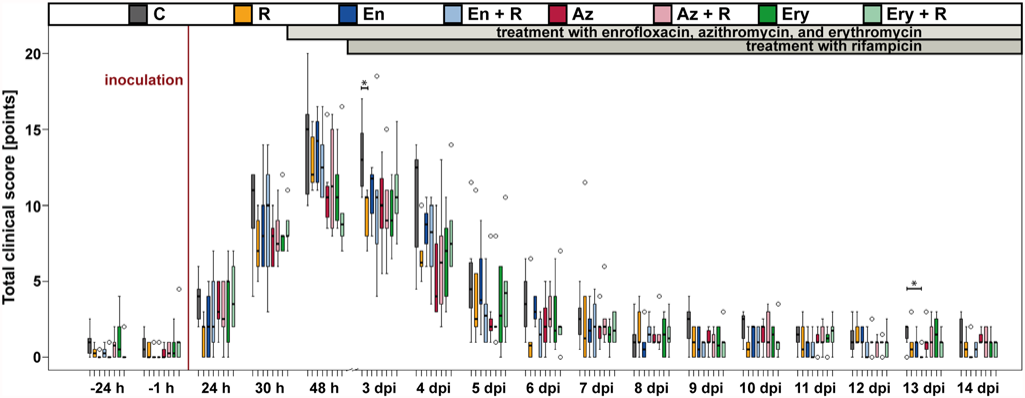
Total clinical score. Following inoculation with *Chlamydia psittaci*, all animals developed signs of an acute respiratory illness, which were maximal 48 hours to 3 days after inoculation. Regardless of treatment, the total clinical score dropped back to baseline level within seven days the latest. Only on two occasions (3 dpi and 13 dpi) there were differences between treated groups and untreated controls that could be statistically secured. Significant differences to the untreated control group at the same day are indicated with asterisks (many-to-one comparisons by Gao *et al*. (2008) with Hochberg-adjustment, *P* ≤ 0.05).

In untreated controls, the total clinical score was slightly higher than in all other groups, but this was already visible before the beginning of antimicrobial treatment. Differences in the total clinical score between untreated controls and other groups were only statistically significant for the rifampicin treated group at 3 dpi (many-to-one comparisons by Gao *et al*. [32] with Hochberg-adjustment, *P* = 0.03) and the enrofloxacin + rifampicin treated group on 13 dpi (*P* = 0.02) (Fig. 2).

**Fig 3:**
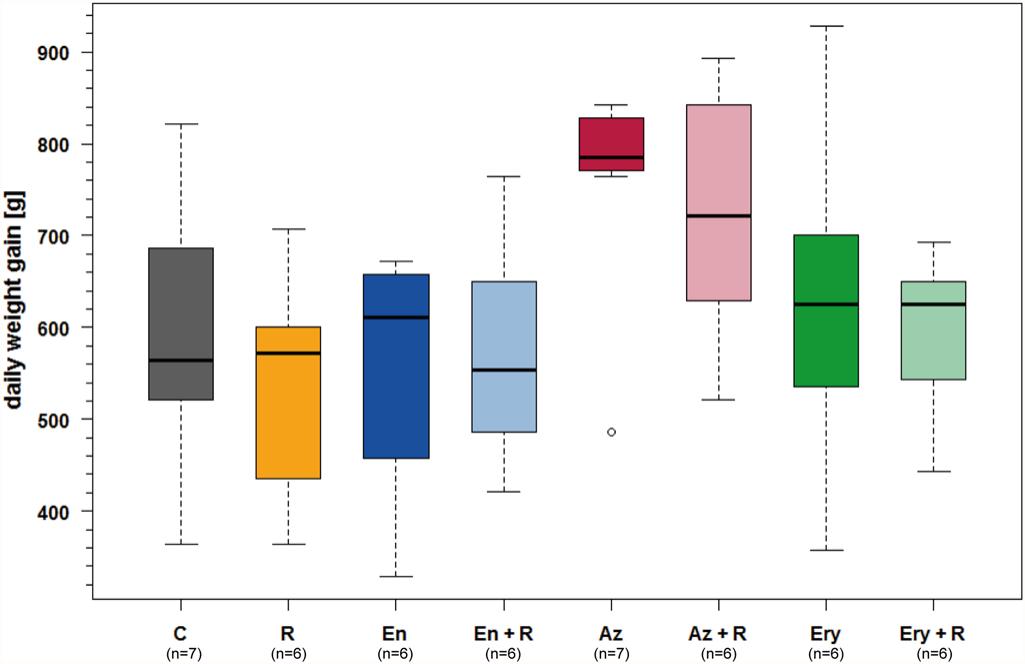
Daily weight gain. Daily weight gain was highest in azithromycin treated animals, but the difference compared to untreated controls was not statistically significant (many-to-one comparisons by Gao *et al*. (2008) with Hochberg-adjustment, *P* > 0.47). C: untreated controls, R: rifampicin, En: enrofloxacin, En + R: enrofloxacin + rifampicin, Az: azithromycin, Az + R: azithromycin + rifampicin, Ery: erythromycin, Ery + R: erythromycin + rifampicin.

The daily weight gain during the study was highest in azithromycin treated groups, but the difference was not statistically significant when compared to untreated controls (many-to-one comparisons by Gao *et al*. [32] with Hochberg-adjustment, *P* > 0.47) (Fig. 3).

### Side effects of treatment

Animals that had received subcutaneous injections of erythromycin during the study developed palm-sized, painful swellings at the injection sites (thorax and neck) that lasted for several days. Injections had to be applied after feeding, since the animals refused milk intake after the injections due to pain. At the injection sites, extensive coagulation necrosis of connective tissue and musculature was seen at necropsy, which was demarcated by granulation tissue and surrounded by edema.

The other treatment regimens were without side effects, no adverse effects on gut microbiome were observed since treated animals did not develop diarrhea or other digestive disorders.

### Systemic inflammation

#### White blood cells

**Fig 4:**
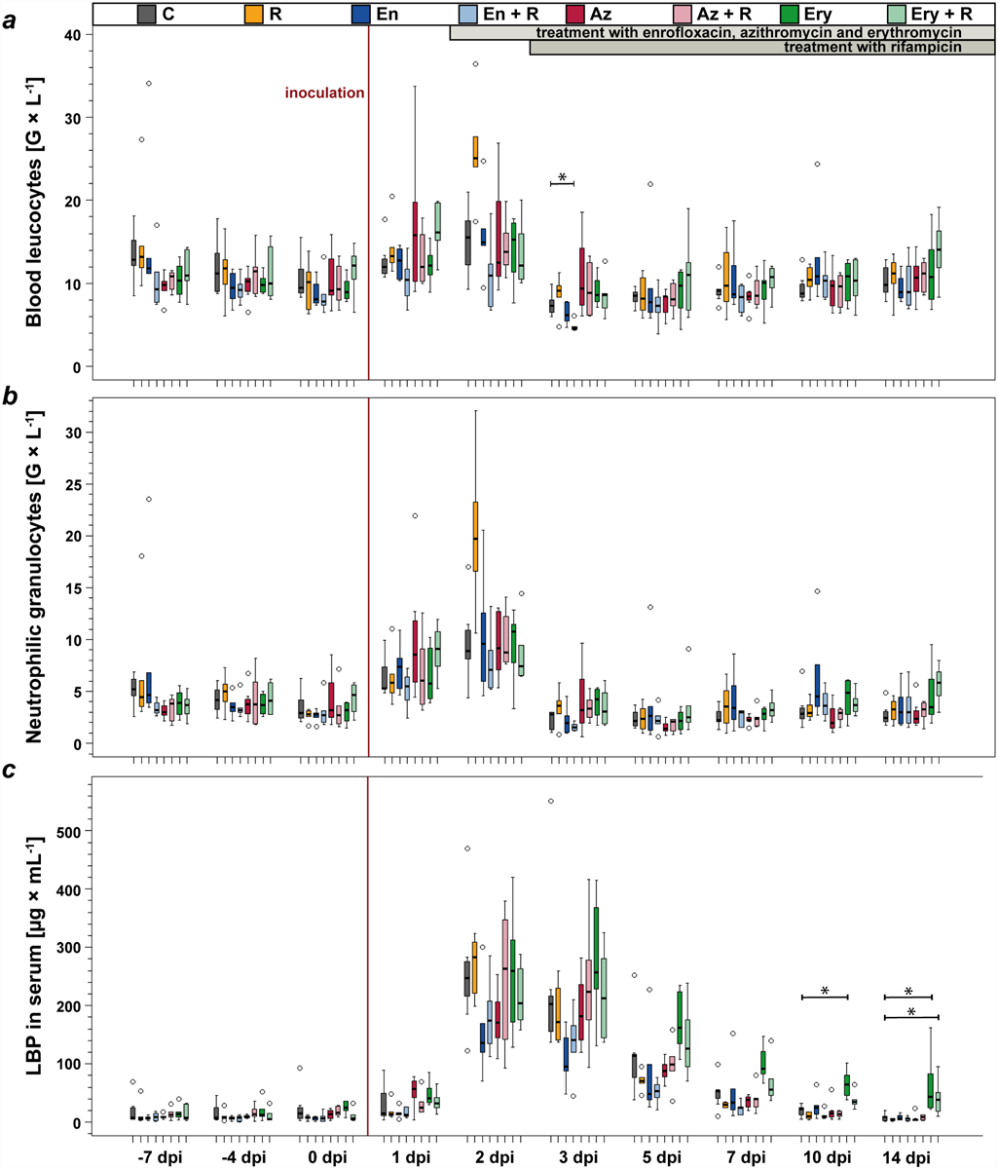
Blood parameters. The day after inoculation with *Chlamydia psittaci*, total blood leucocyte numbers (a) were already markedly increased and numbers stayed above baseline level 2 dpi. The initial increase was followed by a drop slightly below baseline level in all groups. Neutrophilic granulocytes (b) were the cell population being mainly responsible for the changes in absolute leukocyte numbers after inoculation. Other than in the group treated with enrofloxacin + rifampicin on 3 dpi, total leukocyte numbers did not differ significantly between treated and untreated animals, nor did numbers of neutrophilic granulocytes.

The total number of blood leukocytes was increased 1.7-fold within two days after inoculation in all calves. Values dropped to 80% of baseline level the next day, 3 dpi, and baseline values were reached again at 14 dpi. Statistically significant differences between treated and untreated animals were only present for the enrofloxacin + rifampicin treated group on 3 dpi (many-to-one comparisons by Gao *et al*. [32] with Hochberg-adjustment, *P* = 0.009). On 2 dpi, the rifampicin treated group showed higher numbers of total blood leukocytes and neutrophilic granulocytes than other groups (not statistically different when compared to untreated control group), but these values were measured before the first application of rifampicin, therefore a treatment effect as an explanation for this phenomenon can be ruled out (Fig. 4a).

Percentage of neutrophilic granulocytes (segmented and banded forms) increased 2-fold until 2 dpi while total numbers increased 4-fold. Both percentage and total numbers declined to 80% of initial values until 5 dpi and then increased again to be about 1.4-fold of baseline level from 7–14 dpi (Fig. 4b). The percentage of lymphocytes decreased by half within two days after inoculation, leading to a reduction of total lymphocyte numbers to 75% of the initial values. Both, absolute numbers and percentages of lymphocytes were at baseline level at 5 dpi, were they remained until the end of the study. Monocytes increased in both, absolute numbers and percentage of peripheral blood leukocytes, reaching a maximum of 2-fold increase at 5 dpi and levels remaining increased until 14 dpi.

#### Acute phase reaction

Inoculation with *C*. *psittaci* led to an increase in lipopolysaccharide binding protein (LBP) concentration in the peripheral blood of all animals. LBP-levels were maximal at 2 dpi, ranging from 2-fold to 166-fold (median: 19-fold) compared to the levels measured directly before inoculation. In animals of all groups except for the two erythromycin treated groups, LBP concentration in the blood returned to pre-inoculation values by 10 dpi. In animals receiving subcutaneous injections of erythromycin, LBP levels where higher than in the untreated control group from 5 dpi on. For the group treated with only erythromycin, this increase was significant 10 dpi (many-to-one comparisons by Gao *et al*. [32] with Hochberg-adjustment, *P* = 0.03) and 14 dpi (*P* = 0.03) and for animals treated with erythromycin and rifampicin on 14 dpi (*P* = 0.04) (Fig. 4c).

Levels of lipopolysaccharide binding protein (LBP) as a marker of inflammation (c) reached maximal numbers 2 and 3 dpi in all animals, meaning a delay of 24 hours compared to numbers of blood leukocytes and neutrophilic granulocytes. Kinetics of LBP-levels were the same as of the clinical score, baseline level were reached at 10 dpi. In enrofloxacin treated animals, levels of LBP showed a lesser increase than in the untreated control group, but this difference could not be statistically secured. In erythromycin treated animals, LBP levels remained increased until the end of the study; this was statistically significant in comparison to the untreated control group on 10 and 14 dpi.

Significant differences to the untreated control group at the same day are indicated with asterisks (many-to-one comparisons by Gao *et al*. (2008) with Hochberg-adjustment, *P* ≤ 0.05).

C: untreated controls, R: rifampicin, En: enrofloxacin, En + R: enrofloxacin + rifampicin, Az: azithromycin, Az + R: azithromycin + rifampicin, Ery: erythromycin, Ery + R: erythromycin + rifampicin. dpi: days post inoculation.

### Pulmonary inflammation

#### BALF cytology

**Fig 5:**
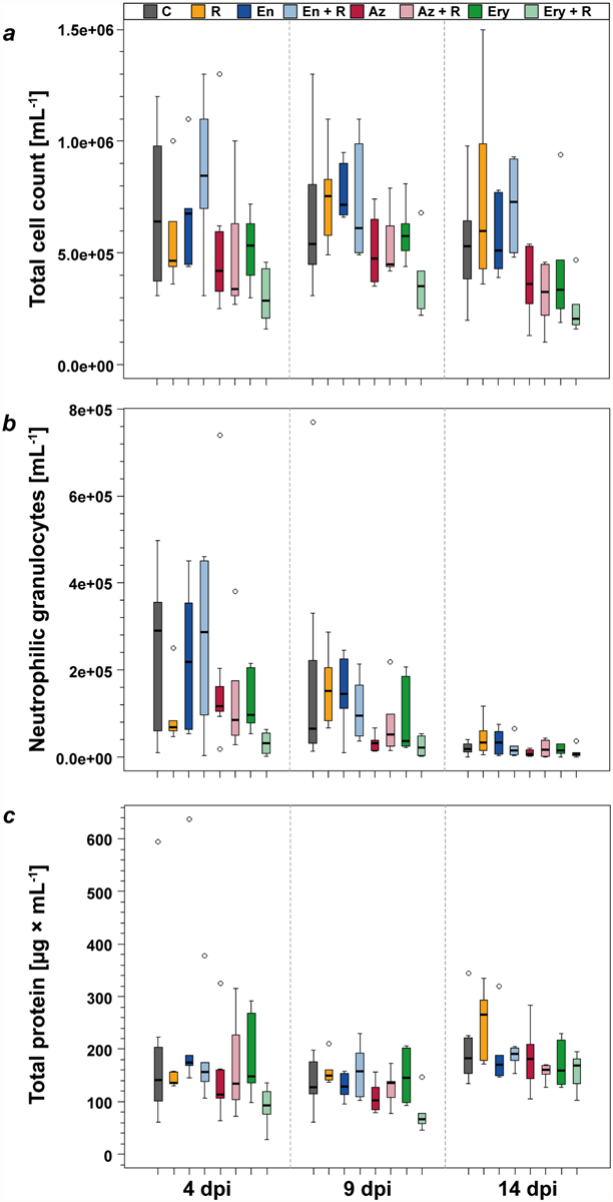
Bronchoalveolar Lavage Fluid (BALF). In the BALF, total cell count (a) remained within comparable limits at all examined time points in all groups. Numbers of neutrophilic granulocytes per mL (b) were highest 4 dpi and dropped continually until the last day of the study. Total protein concentration in BALF-supernatant remained constant throughout the whole study. Statistically significant differences between treated and untreated animals could neither be detected for protein levels nor for numbers of cells or neutrophils per mL in the BALF (many-to-one comparisons by Gao *et al*. (2008) with Hochberg-adjustment, *P* > 0.05). C: untreated controls, R: rifampicin, En: enrofloxacin, En + R: enrofloxacin + rifampicin, Az: azithromycin, Az + R: azithromycin + rifampicin, Ery: erythromycin, Ery + R: erythromycin + rifampicin. dpi: days post inoculation.

Cell count per mL BALF remained relatively constant throughout the study from 4 dpi (5.8e+05; 1.6e+05–1.3e+06) to 9 dpi (6.1e+05; 2.2e+04–1.3e+06) and 14 dpi (4.9e+05; 1.0+05–1.5e+06) (Fig. 5a). Neutrophil numbers per mL BALF (Fig. 5b) and percentage of neutrophils (data not shown) were maximal at 4 dpi (1.7e+05; 2.2e+03–7.4e+05) and decreased continually until 14 dpi (2.2e+04; 0.0e+00–1.2e+05). Neither cell count per mL BALF nor number of neutrophilic granulocytes per mL BALF nor percentage of neutrophils in the total amount of BALF cells differed significantly between treated animals and the untreated control group at any time. However, as shown in Fig. 5a and b, in macrolide treated animals absolute cell count and neutrophilic granulocytes were slightly reduced compared to animals of all other groups, but not significantly lower than in untreated controls (many-to-one comparisons by Gao *et al*. [32] with Hochberg-adjustment, *P* > 0.14).

#### Concentrations of total protein in BALF

The concentration of total protein in the BALF supernatant remained constant from 4 dpi (170 μg/mL; 28–638) until 14 dpi (187 μg/mL; 103–345). There was no significant difference in BALF protein concentration between treated animals and the untreated control group (many-to-one comparisons by Gao *et al*. [32] with Hochberg-adjustment, *P* > 0.09) (Fig. 5c).

### Pulmonary lesions

**Fig 6:**
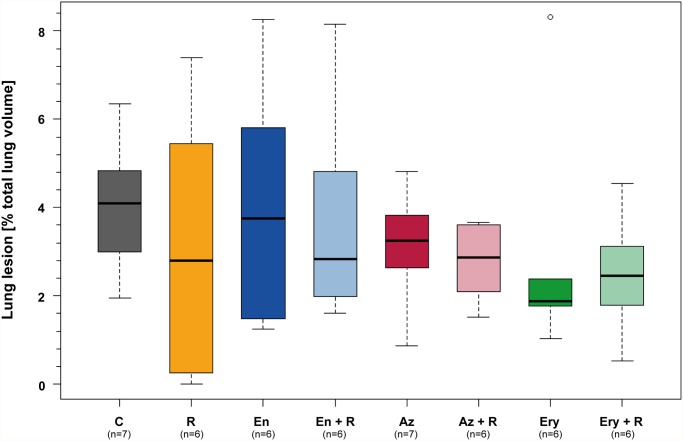
Percentage of altered lung tissue on total lung volume assessed 14 dpi at necropsy. The proportion of altered lung tissue 14 days after inoculation with *C*. *psittaci* did not differ between treated and untreated animals (many-to-one comparisons by Gao *et al*. (2008) with Hochberg-adjustment, *P* > 0.58). C: untreated controls, R: rifampicin, En: enrofloxacin, En + R: enrofloxacin + rifampicin, Az: azithromycin, Az + R: azithromycin + rifampicin, Ery: erythromycin, Ery + R: erythromycin + rifampicin. dpi: days post inoculation.

At necropsy 14 dpi, foci of bronchopneumonia and pleuritis were seen in all calves except for one rifampicin treated animal which showed no lesions. Lung alterations were corresponding to the sites where the inoculum had been applied, i.e. in the caudal part of the left and right apical lobes, the medial lobe and the basal lobes. Necrotic centers and demarcation by fibrous connective tissue were characteristic for the circumscribed pulmonary lesions. The extent of lesions ranged between 0.0% and 8.3% (median: 3.0%) of pulmonary tissue in all animals without significant differences between the treated and untreated animals (many-to-one comparisons by Gao *et al*. [32] with Hochberg-adjustment, *P* > 0.58) (Fig. 6).

Histological examination revealed fibrinous bronchopneumonia in variable degrees of organization characterized by multifocal necrosis, infiltration with lympho-plasma-histiocytic inflammatory cells, hyperplasia of alveolar type 2 epithelial cells, bronchiolitis obliterans, perivascular fibrosis, fibrosis of interlobular septae and ectopic lymphoid follicles. In the majority of calves regeneration predominated. The ratio between zones of necrosis and regeneration varied, however, between individual animals and was not related to treatment groups. In treated animals, chlamydial inclusions were detected by immunohistochemistry (IHC) in 40 calves as small granular labeling in areas with necrosis and few large inclusions in macrophages in zones of regeneration. One of the three calves where chlamydial inclusions were not found by IHC had no pulmonary lesions and the other two calves had small zones of progressed pulmonary regeneration. Two of these calves had been treated with rifampicin only and one with azithromycin and rifampicin.

## Discussion

### Study design

Enrofloxacin, erythromycin, and azithromycin have been used to treat chlamydial infections for years. To address both aspects of veterinary and human medicine, different antimicrobial substances from the family of quinolones and macrolides were chosen: Enrofloxacin is a quinolone commonly used in the field of veterinary medicine, whereas its active metabolite ciprofloxacin is frequently used to treat bacterial infections in human patients. In the class of macrolides, we chose one substance available for the use in animals, i.e. erythromycin and a corresponding drug currently used for treatment of chlamydial infections in humans, i.e. azithromycin. In this study, all drugs were administered alone to assess their effect in treatment of an acute respiratory *C*. *psittaci* infection, and in combination with rifampicin to evaluate whether the increased antichlamydial activity found in the cell culture model by Wolf and colleagues [27] could be reproduced in the living host. One group of animals was treated with rifampicin alone to reveal effects induced only by rifampicin. The calves used in this study had approximately the same weight as adult humans; therefore the rifampicin dose commonly used to treat adult humans was used (600 mg/day per animal).

Treatment was initiated at 30 hours after inoculation. At this time point, clinical signs were beginning to become severe. In a clinical setting, this would be the point to start antimicrobial treatment.

### Antibiotics

Enrofloxacin is a fluorquinolone antibiotic with bactericidal activity by inhibiting bacterial DNA gyrase and thereby preventing DNA supercoiling and DNA synthesis. Fluorquinolones reach high intracellular concentrations and accumulate in bronchial secretions [33]. Enrofloxacin is metabolized to its active metabolite ciprofloxacin, which itself is available as a drug in human medicine, whereas enrofloxacin is only available for veterinary use. Both, enrofloxacin and ciprofloxacin have a broad antimicrobial activity against gram-positive and gram-negative bacteria, except for anaerobic microorganisms and are considered reserve antibiotics [34].

Azithromycin and erythromycin belong to the class of macrolides. This class of antimicrobial substances inhibits bacterial protein synthesis by covalently binding to the 50S ribosomal subunit and is effective mainly against gram-positive microorganisms [35].

Erythromycin is the oldest member of the macrolide family and is frequently used to treat a variety of bacterial diseases in veterinary medicine. It is also available for the use in human patients. Depending on its concentration and on the sensitivity of the microorganism, erythromycin is either bactericidal or bacteriostatic [36].

Azithromycin is superior to erythromycin in terms of pharmacokinetic profile, gastric acid stability, half-life, and bactericidal activity. Tissue concentrations of azithromycin exceed serum concentrations by far and its amphiphilic properties allow it to enter cells and tissue rapidly. It is concentrated in lysosomes of host defence cells, which is the place where chlamydiae replicate. Azithromycin is a valuable tool in the treatment a variety of bacterial infections in humans, including genital tract infections with *C*. *trachomatis*, since in most cases a single dose is sufficient for therapy (reviewed in [37]). A recent meta-analysis reported similar cure rates for azithromycin and doxycycline in treatment of human *C*. *trachomatis* infections. Currently, the use of azithromycin in food-producing animals is banned in the European Union.

Rifampicin, a semi-synthetic antimicrobial drug, enters leukocytes and tissues very well due to its lipid solubility [38,39]. It exhibits bactericidal activity against many gram-negative and most gram-positive bacteria through inhibiting DNA-dependent RNA polymerase [40,41]. In human patients, rifampicin is widely used for the treatment of brucellosis and mycobacterial infections. Yet there is a potential of quickly developing resistance, therefore rifampicin must always be administered in combination with another antimicrobial drug. In experimental settings, rifampicin has been found useful for the treatment of mycobacterial infections in ruminants [42], but in practical settings this would contravene current legislation on authorized substances for the use in food-producing animals in the European Union.

The plasma concentration of ciprofloxacin measured in this study was about 25 to 50% of the plasma concentration of enrofloxacin. This is as expected and reported previously [43]. The MIC of 0.25 μg/mL described for *C*. *psittaci* isolates from turkeys [44] was reached in plasma from 3 dpi to 5 dpi in all calves treated with enrofloxacin. Afterwards, plasma levels of enrofloxacin in some animals were below 0.25 μg/mL. In the lung of animals treated with enrofloxacin, tissue levels of enrofloxacin alone were often lower than 0.25 μg/mL, but this does not take into account the antichlamydial activity of ciprofloxacin. Here, animals with lower lung tissue levels did not show higher clinical scores or more positive reisolation results than animals with higher lung tissue levels. Nevertheless, studies on enrofloxacin treatment of chlamydiae in koalas, cats and pigs revealed that therapeutic levels of the drug are often not reached [25] and that shedding of the pathogen can still occur during [26] or after the end of treatment [45]. Chlamydiae can develop resistance against fluorquinolones when exposed to subtherapeutic dosages over a longer time period [46,47].

The MIC for the *C*. *psittaci* strain DC15 for macrolides and rifampicin reported from *in vitro* studies were between 0.016 and 0.08 μg/mL [27]. These values were reached in plasma and lung tissue of animals treated with macrolides and/or rifampicin with only very few exceptions on single days. As expected, azithromycin levels in lung tissue were by far higher than plasmatic levels.

Macrolides do not only possess antimicrobial, but also anti-inflammatory properties that have been known for decades [48]. They suppress lung defence mechanisms by decreasing neutrophil adhesion and increasing apoptosis of neutrophils, this is why they are successfully used in the maintenance treatment of a variety of chronic airway diseases in humans (reviewed in [49] and [50]). The same effect was shown in foals; erythromycin treatment reduced neutrophil influx into the lung after lavage [51].

In the present study in *C*. *psittaci* infected calves, absolute cell count and numbers of neutrophils in BALF were slightly reduced in macrolide-treated animals as compared to the untreated control group, but this difference could not be secured statistically. Possibly this is due to the fact that macrolide treatment started 30 h post inoculation, leaving enough time for migration of macrophages and neutrophils to inflamed lung areas. Regarding the parameters observed we could not show a significant impact on lung defence mechanisms by erythromycin at a dose of 12 mg/kg bw/day and by azithromycin at a dose of 6 mg/kg bw/day in calves.

### Chlamydiae + antibiotics

There are numerous clinical studies on antimicrobial treatment of chlamydial infections in humans [52–60], but many of them are not placebo-controlled and success of treatment is defined by the eradication of clinical signs rather than the elimination of the pathogen. In studies performing pathogen detection after azithromycin treatment, DNA of *C*. *trachomatis* was still detectable in the urinary tracts of human patients [14,61] and in tissue of experimentally infected mice [62,63]. In the present study, despite considerably high antibiotic levels in the plasma measured at 3 and 5 dpi, reisolation of living *C*. *psittaci* was possible from samples obtained 4 dpi in animals of all groups. The fact that living chlamydiae could be isolated 9 and 14 dpi from control animals, but not from treated animals (except for one animal from the Ery + R group 9 dpi) indicates that the applied substances inhibit the growth of *C*. *psittaci* in the living host, but only after more than 3 days of treatment, thus confirming the used strain DC15 to be sensitive against macrolides, quinolones and rifampicin, as reported by Wolf and colleagues [27].

Even though we could show that treatment with macrolides and quinolones alone or in combination with rifampicin inhibited proliferation of *C*. *psittaci* in the living host, we could not detect any impact on parameters of clinical illness, severity of local and systemic inflammation, or on the number of genome copies detectable in tissue at 14 dpi. Statistically significant differences between treated and untreated groups in the clinical score, numbers of blood leukocytes and neutrophilic granulocytes were not assumed to be of biological relevance, since they occurred only sporadically.

From these facts, one can assume that, in this particular model, it did not matter to the host whether the pathogen was able to replicate or not, because host immune defense mechanisms alone were sufficient to cope with the infection.

There are plenty of reports on mild or subclinical infections with *C*. *psittaci* in humans [64–68] and of asymptomatic *C*. *trachomatis* infections in women [69,70] that resolved without antimicrobial treatment.

Similar findings were reported from studies on subclinical pulmonary abscesses caused by Rhodococcus equi in foals, where spontaneous healing could not be accelerated by adequate antimicrobial treatment [71,72].

These reports are very similar to the results of the present study as neither clinical outcome nor extent of lung lesions differed between treated and untreated animals. Increased LBP response in erythromycin-treated animals in comparison with controls is thought to be due to the inflammatory reaction at the injection site. Apart from this side effect, the LBP response did not differ significantly between treated and untreated animals, thus indicating a comparable severity of inflammation regardless of antimicrobial treatment. Taking all findings together, this leads to the conclusion that the beginning of the treatment in this study was too late to have a significant influence on the course of inflammatory lung disease in the animals, i.e. clinical signs, elevated LBP levels, increased total cell count and neutrophils in blood and BALF. The severity of lesions due to inoculation was already determined at that time point, and everything observed from thereon was the course of remission. As the course of disease in control animals showed, the immune response of the animal sufficed to restore the absence of clinical and inflammatory signs by the end of the study, since clinical score, LBP-levels and blood count reached baseline levels at 10 dpi at the latest. The fact that viable chlamydiae could be reisolated more often and at later time points from control animals than from treated animals did obviously not influence any other parameters that we analysed.

The question whether macrolides or quinolones are adequate for treatment of chlamydial infections in calves cannot be answered by this study. For further studies it would be interesting to screen animals for shedding of chlamydiae after the end of treatment to find out whether treated or untreated animals could clear the infection. The problem with this approach is that shedding of chlamydiae does not occur on a regular basis in infected individuals as could be shown in this and former studies with the used bovine model [13,29] and in a study with *C*. *trachomatis* infected human patients [16].

The fact that chlamydial infections can only be detected when the pathogen is actually shed by the host or when the host becomes seropositive very much limits research in this field, since both are not necessarily true for all individuals infected.

Having in mind that antimicrobial treatment of *Chlamydia*-infected animals is very likely to be without benefit to the host, development of vaccines could be an alternative strategy to limit chlamydial infections in cattle. Currently, available vaccines against chlamydial components of diseases in animals are: abortion in small ruminants (*C*. *abortus*), respiratory disease complex in turkeys (*C*. *psittaci*), and conjunctivitis and respiratory infections in cats (*C*. *felis*). Sterile immunity cannot be expected, a study on *Chlamydia-*associated subclinical mastitis in dairy cows revealed that although bovine mastitis was decreased after vaccination, chlamydiae were still shed into the milk [73].

### Recommendations

Due to the very painful side effects at the injection site, subcutaneous application of erythromycin cannot be suggested for long-term treatment. These adverse reactions at the injection site are known side effects and are reported to last for no longer than 6 days [74]. When using enrofloxacin for treatment of chlamydial infections, the dose must be adapted to reach plasma and tissue levels that are higher than the MIC of the pathogen to enable eradication of chlamydiae and avoid the development of antimicrobial resistance.

### Combination with rifampicin

It has not been possible to extrapolate the findings from the cell culture model, i.e. increased antichlamydial activity of macrolides and quinolones when applied together with rifampicin, to the situation in the living host. This is mainly due to the fact that the host alone was able to cope with the induced infection and treatment itself did not have any measurable influence in terms of host reaction. Reisolation of the living pathogen was possible to the same extent in animals treated with a single drug or the same drug in combination with rifampicin, indicating that the additional application of rifampicin in the living host did not increase the antichlamydial effect of enrofloxacin, erythromycin or azithromycin. On the contrary, the only treated animal in which reisolation was possible 9 dpi had received erythromycin in combination of rifampicin, which does not support the thesis that the combination of erythromycin and rifampicin is particularly effective against chlamydiae in the living host.

### Conclusion

Our results show that treatment of *Chlamydia*-infected calves with enrofloxacin, erythromycin and azithromycin alone or in combination with rifampicin could reduce the number of viable pathogens to the same extent, thus rejecting the hypothesis that combined treatment with rifampicin is superior to single-drug therapy with macrolides or quinolones in the living host. Nevertheless, these findings had no influence on severity of disease and time course of its resolution, as assessed by clinical and pathological findings as well as inflammatory parameters. We can conclude that in this study, a carefully selected and effective antimicrobial treatment in an acute infection was without benefit, since the host’s immune defense mechanisms were sufficient to cope with the infection and all animals, regardless if treated or not, regained clinical health by the end of the study.

## Materials and Methods

### Ethics statement

This study was carried out in strict accordance with the German Animal Welfare Act. The protocol was approved by the Committee on the Ethics of Animal Experiments and the Protection of Animals of the State of Thuringia, Germany (Permit Number: 04–004/11). All experiments were conducted in a containment of biosafety level 2 under supervision of the authorized institutional Agent for Animal Protection. Bronchoscopy was strictly performed under general anesthesia. During the entire study, every effort was made to minimize suffering.

### Animals

Fifty male conventionally raised calves (Holstein-Friesian) were included in this prospective and controlled study. The farm the animals were purchased from did not have any history of *Chlamydia*-associated health problems. In advance, the herd of origin was randomly checked for chlamydial antigen and antibodies by *Chlamydiaceae*-specific PCR and indirect ELISA, respectively, at the National Reference Laboratory for Chlamydiosis. Calves were purchased at the age of 12 to 30 days weighing between 43.6 and 77.6 kg (58.9 ± 8.9; mean ± SD). Animals were included in the study after a quarantine period of at least 21 days and confirmation of a clinically healthy status.

Animals were reared under standardized conditions (room climate: 18–20°C, relative humidity: 60–65%) throughout the entire study. Animal housing was in accordance with the guidelines for animal welfare set forth by the European Community. Nutrition included commercial milk replacers and coarse meal. Water and hay were supplied *ad libitum*. None of the given feed contained antibiotics.

### Study design

All animals were inoculated with 10^8^ inclusion forming units (ifu) of *C*. *psittaci* strain DC15 at the age of 33–53 days. Preparation of the challenge strain was described elsewhere [31]. Eight mL of the *Chlamydia*-containing inoculum were applied intrabronchially and intranasally as described previously [75]. The treatment groups were age matched, and weight of the animals at the time of inoculation (71.7 kg ± 8.6 kg, mean ± SD) did not differ significantly between the groups (Kruskal-Wallis test, *P* = 0.99).

Forty-three calves underwent antimicrobial treatment, whereas 7 calves served as untreated controls (C). The seven treatment groups were treated according to the following regimens: Rifampicin (n = 6): 600 mg rifampicin/day (Eremfat i.v. 600 mg, Riemser Arzneimittel AG, Greifswald, Germany);

Enrofloxacin (n = 6): initially (first treatment) 7 mg/kg bw/day, then (second treatment and following treatments) 5 mg/kg bw/day enrofloxacin (Baytril 10%; Bayer Vital GmbH, Leverkusen, Germany);

Enrofloxacin + Rifampicin (n = 6): initially 7 mg/kg bw/day, then 5 mg/kg bw/day enrofloxacin plus 600 mg rifampicin/day;

Azithromycin (n = 7): initially 10 mg/kg bw/day, then 6 mg/kg bw/day azithromycin (Zithromax Trockensaft 1500 mg, PFIZER PHARMA GmbH, Berlin, Germany);

Azithromycin + Rifampicin (n = 6): initially 10 mg/kg bw/day, then 6mg/kg bw/day azithromycin plus 600 mg rifampicin/day;

Erythromycin (n = 6): 12 mg/kg bw/day erythromycin (Erythrocin Vet. 200mg/ml, CEVA Tiergesundheit GmbH, Düsseldorf, Germany).

Erythromycin + Rifampicin (n = 6): 12 mg/kg bw/day erythromycin plus 600 mg rifampicin/day.

**Fig 7:**
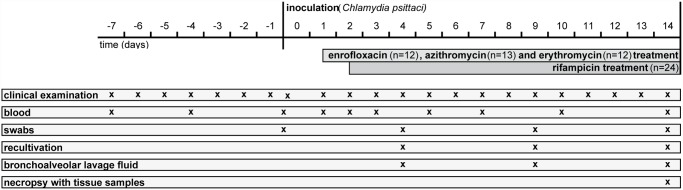
Study design.

Azithromycin was applied orally with the milk replacer once daily starting 36 hours after inoculation (pi). Rifampicin was administered intravenously in 500 mL isotonic saline solution once daily over 30 minutes starting 48 hours pi. Enrofloxacin and erythromycin were injected subcutaneously once daily, starting 30 hours pi. Treatment continued until 13 days pi (dpi), and all animals were euthanized and necropsied 14 dpi. Sampling throughout the study is illustrated in Fig. 7.

The two veterinarians performing the treatments and monitoring the animals were the only persons who knew the assignment of the animals to the different treatment groups. Variables assessed *in vivo* (body temperature, respiratory rate, heart rate) can be regarded as ‘objective’ since they are measured values. An individual ID according to the rules of our institute’s quality management system was given to all specimens examined *ex vivo*. Lab investigators were therefore disabled to identify individual animals or treatment regimens.

### Clinical Scoring and daily weight gain

Clinical examination of all animals was performed twice daily, starting 4 days before challenge. A previously described scoring system was used to summarize the observations [31]. For determination of daily weight gain, each animal was weighed directly before inoculation and directly before necropsy. The difference was calculated and divided by the number of days in between.

### Collection of swabs and blood and blood analyses

Nasal and fecal swabs were taken one hour before inoculation and immediately before necropsy. Pharyngeal and fecal swabs were obtained 4 dpi and 9 dpi. Swabs were sampled as described previously [13].

Venous blood of all animals was drawn from the *Vena jugularis* 7 days, 4 days and 1 hour prior to inoculation and 1 dpi, 2 dpi, 3 dpi, 5 dpi, 7 dpi, 10 dpi, and 14 dpi (Fig. 7). After the beginning of antimicrobial treatment, blood was always drawn 18 hours after treatment with enrofloxacin, azithromycin or erythromycin and 21 hours after treatment with rifampicin.

Collection of blood, white blood cell count, differentiation of white blood cells, serum preparation, and quantitative detection of LBP with an enzyme-linked immunosorbent assay (ELISA) were described previously [29,30].

### Necropsy, pathological evaluation, collection of tissue samples, histology, and immunohistology

Euthanasia of the calves and exenteration of the lung was performed as described before [31]. Pathological evaluation of lung lesions and tissue sampling as well as histology and immunohistology has been described in detail [13].

### Collection of bronchoalveolar lavage fluid (*in vivo*, *post mortem*) and BALF analyses

Endoscopic sampling of BALF in the anesthetized animal was performed 4 and 9 dpi as described previously [75]. At 14 dpi, BALF was obtained from freshly exenterated lungs immediately after exsanguination as described in [13]. Isotonic, sterile, body-warm saline was always used as flushing liquid. The obtained BALF was immediately collected in siliconized glass bottles and stored on ice until further preparation.

BALF recovery *in vivo* was 79.7 ± 4.5% (mean ± SD); *post mortem* it was 63.1 ± 7.2% (mean ± SD). Both values did not differ significantly between the treatment groups (Kruskal-Wallis test, *P* > 0.13).

Preparation of BALF, BALF cell count and differentiation were done as described previously [13]. Analysis of total protein in BALF supernatant was also described elsewhere [31].

### Collection of bronchial brushings

Bronchial brushings for the reisolation of the chlamydiae were obtained 4 and 9 dpi during bronchoscopy from the right lung, just caudal of the *Bifurcatio tracheae* as described previously [75]. The brush was rinsed thoroughly in stabilizing SPGA medium (containing sucrose, phosphatile substances, glucose and bovine albumin [76]). Samples obtained at 4 dpi were processed immediately, whereas samples obtained at 9 dpi were stored at -20°C until further use.

### Reisolation

Isolation of *C*. *psittaci* in buffalo green monkey kidney cells was performed according to standard procedures. To confirm viability of the pathogen, five randomly selected culture positive samples were subjected to further passaging and 11 samples were subjected to DNA-sequencing. Samples were bronchial brushings obtained 4 and 9 dpi and altered lung tissue collected at necropsy (14 dpi).

### Detection of chlamydial DNA using quantitative real-time PCR (qrt-PCR) and sequencing

Samples of macroscopically altered and macroscopically normal lung tissue, mediastinal lymph node, swabs (pharyngeal, nasal, faecal), venous blood, and selected samples of reisolated and passaged chlamydiae were examined for chlamydial DNA. Extraction of DNA was performed using the High Pure PCR Template Preparation Kit (Roche Diagnostics, Mannheim, Germany) following the manufacturer’s instructions. In qrt-PCR testing for the family *Chlamydiaceae*, two 1.0 μl aliquots of the final eluate were used as templates [77]. Ct values of less than 38.0 in both replicates measured were considered positive.

Additional testing for the species of *C*. *psittaci* was performed for the swabs and blood samples [78]. Passaged chlamydiae that were reisolated in cell culture from bronchial brushings and lung tissue were subjected to sequencing of the complete *ompA* gene to confirm the identity to challenge strain DC15 [79].

### Antibiotic levels

Antibiotic levels of azithromycin, erythromycin and rifampicin were determined in plasma obtained 1 h prior to inoculation as well as 3, 5, 7, 10, and 14 dpi and in tissue samples collected at necropsy 14 dpi (unaltered lung, liver and muscle). The samples were all stored at -80°C until analysis and were tested for the presence of the respective drugs that had been administered. Preparation of samples and analysis was performed as follows:

#### Levels of erythromycin, azithromycin and rifampicin in animals treated with macrolide alone or in combination with rifampicin

Two hundred μL of each plasma sample were extracted with 600 μL acetonitrile containing sulfaphenazole as internal standard. After vortex and centrifugation, the supernatant was evaporated in a nitrogen stream until dryness. The extract was resuspended in 500 μL acetonitrile/milliQwater 20/80 and then filtered on polyvinylidene fluoride (PVDF) 0.45 μm.

Pieces of 0.5 g of each tissue sample were shaken with 250 μL milliQwater containing sulfaphenazole as internal standard, and then extracted with 2 mL acetonitrile for 10 min.

After centrifugation, the supernatant was evaporated in a nitrogen stream until dryness. The extract was resuspended in 500 μL acetonitrile/milliQwater 20/80 and filtered on PVDF 0.45 μm.

LC-MS-MS analysis of all samples was done using an Agilent1290 HPLC (Agilent Technologies) coupled with MS-MS detector 6460 (Agilent Technologies). The chromatographic column was Waters BEH C18 (150mm x 2.1mm x 1.7 μm). Five μL of each sample were injected and separated using a gradient from 80% water 0.05% formic acid and 20% acetonitrile to 100% acetonitrile and a flow rate of 0.4 mL/min for 8 min.

For plasma quantification, the calibration was prepared in matrix with spiked plasma from 1 to 200 ng/mL with sulfaphenazole as internal standard.

For tissue quantification, the calibration was prepared in matrix with spiked tissue from 10 to 500 ng/mL with sulfaphenazole as internal standard.

The detection was performed using positive electrospray ionization (ESI+) with the MRM transitions: azithromycin 749/83.2 and 749/158, erythromycin 734/158 and 734/83, rifampicin: 823/791 and 823/399 sulfaphenazole (internal standard): 315/158 and 315/92.

The threshold was set to 3.5 ng/mL for all analytes.

#### Levels of enrofloxacin and rifampicin in animals treated with rifampicin and/or enrofloxacin

Plasma samples were mixed 1 + 9 (v/v) with water. After addition of internal standard of enrofloxacin and ciprofloxacin an aliquot was injected directly into an online solid phase extraction chromatography (OSPE) system while it was in the loading position. Matrix components contained in the injected sample were separated from the retained analytes on an extraction column suited for pre-treatment of samples at high flow rates. After switching to the elution position, the analytes were transferred to an analytical column. The quantitative determination was performed in a tandem mass spectrometric detector (MS/MS).

The limit of quantitation was 25 μg/L for each analyte.

For analysis of tissue samples, the entire sample material (between 0.4 and 1.4 g) was cut into small pieces using a scalpel and then filled into an Ultra-Turrax Tube Drive tube (IKA-Werke GmbH & Co. KG, Staufen, Germany). Enrofloxacin and ciprofloxacin were extracted simultaneously by homogenisation with 15 mL of a mixture of acetonitrile, water and formic acid (500/500/0.1, v/v/v) and subsequent centrifugation.

For the determination of enrofloxacin and ciprofloxacin the extract was diluted 1 + 1 (v/v) with water and internal standard was added. Measurement was performed as described above by OSPE-LC-MS/MS.

For rifampicin analysis the extract was diluted 1+1 (v/v), for liver 1 + 9 (v/v), with acidic water by HPLC with tandem mass spectrometric detection.

The limit of quantification was 100 μg/kg for enrofloxacin and ciprofloxacin and 10 μg/kg for rifampicin for all sample materials.

A more detailed description of the analysis of enrofloxacin and ciprofloxacin levels has been published earlier [80].

Antibiotic levels above threshold (animals treated with macrolide alone or in combination rifampicin) or detection level (animals treated with rifampicin and/or enrofloxacin) were considered significant.

### Exclusion of co-infections

The herd the calves originated from was known to be free of bovine herpes virus 1 (BHV-1) and bovine virus diarrhea/mucosal disease virus (BVDV). Immunohistochemical examination of ear biopsies for BVDV antigen [81] was negative indicating that no calves were immunocompromised by persistent BVDV infection.

Routine microbiological screening on the day after purchase revealed that all animals were negative for *Salmonella* infections (fecal swabs). During the quarantine period, all 50 calves included were checked serologically for antibodies against *C*. *abortus* and *C*. *psittaci* (ID SCREEN Chlamydophila abortus indirect multi-species ELISA Kit; ID vet, Grabels, France). All animals were serologically negative prior to inoculation.

The presence of the relevant respiratory co-pathogens *Mycoplasma*, *Pasteurella* or *Mannheimia* spp. was evaluated in nasal swabs taken immediately before challenge and before necropsy, and in samples of lung tissue obtained during necropsy. Neither *Mannheimia haemolytica* nor *Mycoplasma bovis* nor *Pasteurella multocida* were detected in any swab or lung sample. Serology at the beginning of the quarantine period and at 14 dpi was used to check for viral co-pathogens i.e. bovine respiratory syncytial virus (BRSV), parainfluenza 3 virus (PI3), adenovirus type 3, BHV1 and BVDV (Bio-X respiratory penta ELISA Kit, Bio-X-Diagnostics, Jemelle, Belgium). Maternal antibodies were seen against BRSV (42/50), PI3 (46/50), BVDV (26/50), BHV1 (20/50) and adenovirus type 3 (47/50).

To eliminate any unknown bacterial infections, all animals received antibiotic treatment in the week after purchase according to our animal facility’s regulations for the entry of new animals. Calves to be treated with enrofloxacin, enrofloxacin and rifampicin or rifampicin alone and the two co-housed untreated control animals were treated with subcutaneous injections of 10 mg/kg bw/day erythromycin on three consecutive days in the week after purchase. All other animals received subcutaneous injections of 5 mg/kg bw/day enrofloxacin on three consecutive days, beginning two days after purchase.

It has been reported that seven days after subcutaneous injection of enrofloxacin in cattle, residue levels in liver, kidney, muscle, and fat are <10 μg/kg [82]. In calves that had received intramuscular injections of erythromycin, antimicrobial activity in tissue was detected no longer than 3 days after the last injection [74], thus ruling out an influence of pre-study treatment on any results.

### Statistical methods

R [83] was used for statistical evaluation of the data. In addition, the package Plotrix [84] was used for computing the graphs. Due to the small group size of n = 6 and n = 7, data was assumed not to be normally distributed. Kruskal-Wallis test was carried out to compare values of more than two different groups. To compare values of treated groups against values of the untreated control group, the nonparametric multiple tests for many-to-one comparisons by Gao *et al*. [32] with Hochberg-adjustment of the p-value from the package nparcomp [85] was performed. Only significant differences between control group and treated groups are given, differences between treated groups were of no interest in this study. For comparison of two groups, Mann-Whitney U test from the package coin [86] was used. Unless stated differently, data are given as median and range. In ‘Box and Whiskers plots’, outlier values (circles) were more than 1.5 times the length of a box away from the median. Values of *P* ≤ 0.05 were considered significant.
